# Prevalence and characteristics of COPD among pneumoconiosis patients at an occupational disease prevention institute: a cross-sectional study

**DOI:** 10.1186/s12890-018-0581-0

**Published:** 2018-01-29

**Authors:** Yating Peng, Xin Li, Shan Cai, Yan Chen, Weirong Dai, Wenfeng Liu, Zijing Zhou, Jiaxi Duan, Ping Chen

**Affiliations:** 1 Department of Respiratory Medicine, The Second Xiangya Hospital, Central South University, Changsha, Hunan 410011 China; 2 Research Unit of Respiratory Diseases, Central South University, Changsha, Hunan 410011 China; 3 Diagnosis and Treatment Center of Respiratory Disease, Central South University, Changsha, Hunan 410011 China; 4 Hunan Institute of Occupational Disease Prevention, Hunan Provincial Center for Disease Control and Prevention, 410011 Changsha, People’s Republic of China

**Keywords:** Pneumoconiosis, COPD, Prevalence, Risk factors

## Abstract

**Background:**

Pneumoconiosis may play an important role in the development of chronic obstructive pulmonary disease (COPD), and the complication of COPD may impose a heavy burden of illness.

**Methods:**

The study was conducted in Hunan Province in China from December 1, 2015, to December 1, 2016. Consecutive underground male pneumoconiosis patients employed for at least 1 year were recruited from the Hunan Occupational Disease Prevention Institute. Patient information, respiratory symptoms and clinical data were collected using a structured questionnaire. The diagnosis of COPD were assessed using the Global Initiative for Chronic Obstructive Lung Disease (GOLD) criteria. Logistic regression analyses were conducted to examine the clinical and demographic risk factors of COPD among pneumoconiosis patients.

**Results:**

The prevalence of COPD in our sample of pneumoconiosis patients was 18.65% (119/638). In pneumoconiosis patients with and without smoking history, the prevalence of COPD was 19.32 and 16.77%. Compared with non-COPD patients, those with COPD are older in age, have longer exposure time, have lower body mass index (BMI), have a higher smoking index and have worse pulmonary function (all *p* < 0.05). For the five respiratory symptoms (cough, sputum, wheeze, dyspnea, and chest tightness), only the presence of wheeze and the severity scores for wheeze or dyspnea showed significant differences between the COPD and non-COPD groups (*p* < 0.01). Multivariate logistic regression analysis revealed that advanced pneumoconiosis category, older age and the presence of wheeze symptoms were significant risk factors for the development of COPD among pneumoconiosis patients.

**Conclusion:**

Pneumoconiosis patients are at a high risk of COPD, and pneumoconiosis patients with COPD may suffer more severe respiratory symptoms, such as wheeze and dyspnea, than patients without COPD. Advanced pneumoconiosis category, older age and the presence of wheeze symptoms are associated with an increased risk of COPD in pneumoconiosis. We proposed that a routine assessment of lung function is necessary for timely and adequate clinical management.

## Background

Coal or silicosis dust exposure are risks for a range of chronic respiratory diseases, including coal workers’ pneumoconiosis (CWP), silicosis, diffuse dust-related fibrosis, and COPD. Pneumoconiosis is one of the most common occupational diseases, and it is associated with the inhalation of mineral or organic dust [1] and poor personal protection [2]. The destruction of the pulmonary parenchyma and the upper airway is progressive and irreversible throughout the disease, and there is no effective therapy for the disease according to current guidelines [3]. China is one of the world’s leading countries in the number of cases of pneumoconiosis, and the prevalence has remained very high in recent decades. A total of 23,152 new cases of pneumoconiosis were diagnosed in 2013, accounting for 87.72% of all reported occupational diseases in China [4].

COPD is characterized by partial reversible airflow limitation, and it is predicted to be ranked as the third most frequent cause of death in 2020 [5]. In addition to cigarette smoking, genetic factors, longstanding asthma, outdoor air pollution, secondhand smoke exposure, biomass smoke and occupational exposures are also recognized as risk factors for COPD [6–8]. In China, COPD was associated with high exposure to dust or gas/fume with no evidence of effect modification by smoking and the overall population attributable fraction for COPD due to occupational exposure was reported as 10.4% [9]. Moreover, pneumoconiosis was found to be a factor of severity in acute exacerbation of COPD (AECOPD) [10]. Patients admitted for AECOPD complicated with CWP had longer hospitalization times, a higher cost of hospitalization, and higher rates of infective microorganisms in respiratory secretions and/ or blood cultures compared to patients without pneumoconiosis [10]. This study aimed to assess the prevalence and risk factors for developing COPD and to compare the clinical difference between pneumoconiosis patients with and without COPD.

## Methods

### Study objectives and design

The Strengthening the reporting of observational studies in epidemiology (STROBE) checklist was followed with respect to the study design as much as possible [11]. Pneumoconiosis patients who worked underground for at least 1 year were consecutively recruited in the Hunan Occupational Disease Prevention Institute from Dec 2015 to Dec 2016. Detailed questionnaires and comprehensive clinical examinations were conducted for the participants. The study was approved by the ethics review board of the Hunan Occupational Disease Prevention Institute, and written informed consent was obtained from each participant.

Subjects with identified cases of acute pulmonary infection or pneumothorax, pulmonary tuberculosis/intrapulmonary infection, emphysema, chronic bronchitis, asthma, bronchiectasis or pulmonary interstitial fibrosis were excluded from the study. Subjects who were unable to cooperate with lung function tests were also excluded from the study.

### Data collection

Upon participants’ follow-up visits to the institute, medical records were reviewed from the database, and complete check-up procedures were performed for eligible participants. Basic medical information and questionnaires for COPD were collected, and pulmonary function tests were conducted. The interviewer-administered questionnaire (Appendix i) comprised specific questions related to possible risk factors. This information included age, height, weight, smoking status, duration of employment in different professions and workplaces, exposure duration time, past medical history, family history, exposure to silicosis or coal dust, and whether or not air drills were engaged. Cigarette smoking history was collected as the smoking index (SI = pack per day * smoking year) and classified into four types: heavy (≥20 pack-years), moderate (≥10, <19 pack-years), mild (<10 pack-years) and never cigarette smokers. BMI status was classified into below normal (< 18.5 kg/m^2^), normal (18.5~ 23.9 kg/m^2^), overweight (24.0~ 27.9 kg/m^2^), and obesity (≥28 kg/m^2^). The diagnosis of radiography pneumoconiosis was performed according to the China National Diagnostic Criteria [12], which is the same as that of the 1980 International Labour Organization (ILO) in the judgment of opacity profusion. The pneumoconiosis patients were classified into category I, category II, and category III according to the size, profusion, and distribution range of the opacity of the chest X-ray [13]. Pulmonary function was measured using a spirometer MasterScreen™ pulmonary function test (PFT) system (Germany), and spirometric measurements met the standards of the American Thoracic Society and the European Respiratory Society [14]. A portable diffusion carbon monoxide detector was used to measure carbon monoxide diffusion capacity. The diagnosis of COPD was performed according to the update 2015 GOLD guideline [15]. The criterion for the confirmation of a diagnosis of COPD was made through clinical manifestations, risk factors, and a post-bronchodilator forced expiratory volume in the first second/forced vital capacity (FEV_1_/FVC) ratio of < 0.7 on PFT. The level of GOLD spirometric was as follows: FEV_1_% ≥ 80% predicted was designated mild airflow limitation; 50% ≤ FEV_1_ < 80% predicted was designated moderate airflow limitation; 30% ≤ FEV_1_ < 50% predicted was designated severe airflow limitation; and FEV_1_ < 30% predicted was designated very severe airflow limitation.

Respiratory symptoms (cough, sputum, wheeze, dyspnea and chest tightness), as well as the COPD Assessment Test (CAT) [16] and the COPD clinical questionnaire (CCQ) [17], were completed individually. The questions regarding respiratory symptoms are listed as follows: “Do you usually have a cough in the absence of a cold and cough most days, at least three times per week each year? Yes/No”; “Do you have sputum most days, at least three times per week each year? Yes/No”; “Did you have chest wheezing in the past 12 months? Yes/No”; “Do you have shortness of breath when walking faster on flat ground or on a slight slope or walk slower than others your age on flat ground due to shortness of breath? Yes/No”; and “Do you have chest tightness most days, especially after force? Yes/No”. Positive respiratory symptoms were determined when the answer was “Yes”. At the same time, quantitative data on the severity of symptoms were evaluated on a scale from 0 to 5 (severity scale: 0 = Not at all, 1 = Just a little, 2 = Somewhat, 3 = Moderately, 4 = Quite a lot, 5 = Very much).

### Statistical analysis

Statistical analysis was performed using the Statistical Package for Social Sciences 17.0 (SPSS Inc.®, Chicago, IL, USA) software. The continuous variables had a skewed distribution and were expressed as medians (interquartile ranges). Chi-square test analysis was conducted to compare the frequencies between two groups and among four groups. Comparisons of continuous and rank variables were determined by using the Mann-Whitney U test. Correlations between pulmonary functions and scale scores were analyzed using Spearman correlation analysis. Univariate and multivariate logistic regression analysis was used to determine the risk factors of COPD, and the enter method was used to build the statistical model in the univariate regression analysis. ORs with 95% CI are presented. A *p*-value was used to characterize statistically significant results.

## Results

**Fig. 1:**
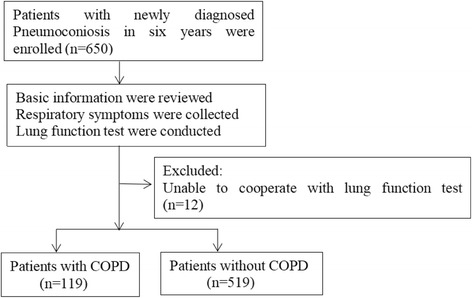
Study flow diagram

A total of 650 male pneumoconiosis patients were recruited during the study period. A total of 12 pneumoconiosis patients were excluded because of their inability to cooperate with lung function tests. Finally, 638 eligible radiography pneumoconiosis patients were included in our study (Fig. 1). The mean age of these 638 subjects was 50.75 years (range 28–80 years), and the mean exposure time was 17.70 years (range 1–38 years).

According to the GOLD guideline, 119 patients (18.65%) had COPD. The prevalence of COPD was 12.32, 22.37, 45.00 and 47.06% in the patients with age of 41–50, 51–60, 61–70 and 71–80 years, respectively. The prevalence of COPD was 41.67, 18.97, 15.26 and 18.18% in the patients with BMI below normal, normal, overweight, and obesity. The prevalence of COPD was 8.24, 13.64 and 46.75% in the patients with category I, category II, category III. The prevalence of COPD was 12.72, 17.54, 27.42 and 11.76% in the patients with exposure time of 0–10, 11–20, 21–30, and 31–40 years, respectively. The prevalence of COPD was 20.42% in patients with silicosis exposure, 18.15% in patients with coal exposure. The prevalence of COPD was 21.89% in patients with air drill and 15.00% in patients without air drill. The prevalence of COPD was 19.32 and 16.77% in patients with and without smoking history and was 23.21%, 7.5%, 17.35% in the patients with heavy, moderate, mild smoking history (Table 1).

**Table 1:** Demographic characteristics of the pneumoconiosis patients and prevalence of COPD

Total (*n* = 638)		Subjects		Prevalence of COPD	
		No.	%	No.	%
Age,years	21–30	2	0.31%	0	0%
	31–40	10	1.57%	0	0%
	41–50	341	53.45%	42	12.32%
	51–60	228	35.74%	51	22.37%
	61–70	40	6.27%	18	45.00%
	71–80	17	2.66%	8	47.06%
BMI	Below normal	24	3.76%	10	41.67%
	Normal	369	57.84%	70	18.97%
	Overweight	190	29.78%	29	15.26%
	Obesity	55	8.62%	10	18.18%
Category	I	352	55.17%	29	8.24%
	II	132	20.69%	18	13.64%
	III	154	24.14%	72	46.75%
Exposure time, years	0–10	173	27.12%	22	12.72%
	11–20	228	35.74%	40	17.54%
	21–30	186	29.15%	51	27.42%
	31–40	51	7.99%	6	11.76%
Exposure type	Silicosis dust	142	22.26%	29	20.42%
	Coal dust	496	77.74%	90	18.15%
Air drill type	No	300	47.02%	45	15.00%
	Yes	338	52.98%	74	21.89%
Smoking history	Never	167	26.18%	28	16.77%
	Mild	98	15.36%	17	17.35%
	Moderate	80	12.54%	6	7.5%
	Heavy	293	45.92%	68	23.21%
Education	Below primary school	8	1.25%	1	12.5%
	Primary school	197	30.88%	43	21.83%
	Middle school	423	66.30%	73	17.26%
	High school	6	0.94%	2	33.33%
	Some college	4	0.63%	0	0%

Compared with non-COPD group, the combined COPD group have significantly higher pneumoconiosis category (*p* = 0.00), older age (*p* = 0.00), longer exposure time (*p =* 0.01), lower BMI (*p* = 0.01), higher SI (*p =* 0.02) and more air drill type (*p =* 0.03). In addition, those with COPD had significantly severe airflow limitation, lower levels of Pre-FEV_1_, Pre-FVC, FEV_1_/FVC, DLCO and DLCO% compared with non-COPD patients (*p* all<0.00). The severity scores in wheeze, dyspnea, CAT, and CCQ were significantly higher in the COPD group than in the non-COPD group (*p* all<0.00). No significant differences were found in the severity scores of cough, sputum and chest tightness symptoms and degree of education between patients with and without COPD (*p* > 0.05) (Table 2).

**Table 2:** Comparison of clinical characteristics between pneumoconiosis patients with and without COPD

Characteristics	Non-COPD (*n* = 519)		COPD (*n* = 119)		*P* Value
	*N*	%	*N*	%	
Pneumoconiosis category					
I	323	62.24	29	24.37	0.00 ^a^,**
II	114	21.97	18	15.13	
III	82	15.80	72	60.50	
Exposure type					
Silicosis	113	21.77	29	24.37	0.54 ^a^
Coal	406	78.23	90	75.63	
Engaged in Air drill					
Yes	264	50.87	74	62.18	0.03 ^a^,*
No	255	49.13	45	37.82	
Age,y	49.00(46.00,53.00)		53.00(48.00,60.00)		0.00 ^b^,**
BMI, kg/m^2^	23.14(21.30,25.22)		22.32(20.10,24.37)		0.01 ^b^,**
Exposure time, y	15.00 (10.00,25.00)		20.00(13.00,25.00)		0.01 ^b^,*
Smoking index	12.50(0.00,27.00)		20.00(1.25,32.00)		0.02 ^b^,*
Education	2.00(1.00,2.00)		2.00(1.00,2.00)		0.25
Category	1.00(1.00,2.00)		3.00(2.00,3.00)		0.00 ^b^,**
Pulmonary function					
Airflow limitation	0.00(0.00,0.00)		2.00(2.00,3.00)		0.00 ^b^,**
Pre-FEV_1_	89.70(81.40,98.60)		57.90(41.60,70.60)		0.00 ^b^,**
Pre-FVC	91.30(83.30,99.40)		76.80(65.40,87.20)		0.00 ^b^,**
FEV_1_/FVC	80.56(75.94,84.55)		62.19(51.84,65.63)		0.00 ^b^,**
DLCO	8.29(7.13,9.44)		6.86(5.42,7.99)		0.00 ^b^,**
DLCO%	91.90(79.20,104.50)		76.40(60.60,88.50)		0.00 ^b^,**
Severity scores					
Cough	1.00(1.00,3.00)		2.00(1.00,3.00)		0.37
Sputum	1.00(1.00,3.00)		2.00(1.00,3.00)		0.39
Wheeze	0.00(0.00,0.00)		0.00(0.00,1.00)		0.00 ^b^,**
Dyspnea	2.00(2.00,2.00)		2.00(2.00,2.00)		0.00 ^b^,**
Chest tightness	2.00(1.00,3.00)		2.00(1.00,3.00)		0.29
CAT	10.00(7.00,14.00)		13.00(8.00,17.00)		0.00 ^b^,**
CCQ	21.00(16.00,26.00)		25.00(21.00,29.00)		0.00 ^b^,**

Note: Airflow limitation are presented as average rating, 1 = mild, 2 = moderate, 3 = severe, 4 = very severe

*p<0.05, **p<0.01

^a^Chi-square test

^b^Mann-Whitney U test

**Fig. 2:**
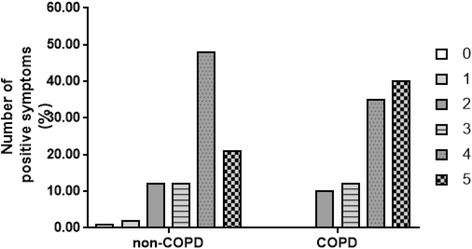
Percent of patients with a certain number of positive respiratory symptoms in pneumoconiosis patients without COPD and with COPD

**Fig. 3:**
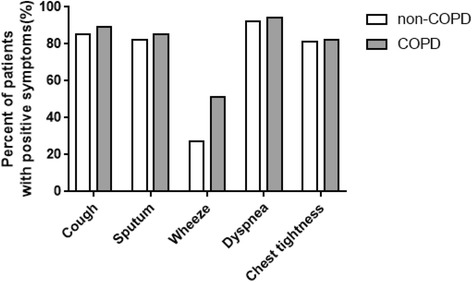
Percent of patients with each positive respiratory symptom in pneumoconiosis patients without COPD and with COPD

The distribution and severity scores of respiratory symptoms between patients with and without COPD were different. In non-COPD patients, the percentages of patients with 0, 1, 2, 3, 4, and 5 positive symptoms were 1.93, 2.70, 12.14, 12.91, 48.36, and 21.97%, respectively. However, in combined COPD patients, the percentage of patients with 0, 1, 2, 3, 4, and 5 positive symptoms was 0, 8.4, 10.92, 12.61, 35.29, and 40.34%, respectively (Fig. 2). In non-COPD patients, the percent of patients have cough, sputum, wheeze, dyspnea or chest tightness symptoms were 85.36, 82.47, 27.36, 92.10 and 81.89%, respectively. In combined COPD patients, the percent of patients have cough, sputum, wheeze, dyspnea or chest tightness symptoms were 89.08, 85.71, 51.26, 94.96 and 82.35%, respectively. Compared with non-COPD patients, COPD patients have significantly higher percentage of positive respiratory symptoms in wheeze (61/119, 50.83% vs 142/519, 27.36%, *p* = 0.00), but not in cough, sputum, dyspnea and chest tightness (Fig. 3).

Severity scores of wheeze were correlated with pre-FEV_1_ (*r* = − 0.41 *p* = 0.00), FEV_1_/FVC (*r* = − 0.28, *p* = 0.00), DLCO (*r* = − 0.16, *p* = 0.08) and DLCO% (*r* = − 0.22, *p* = 0.02). Severity scores of dyspnea correlated with pre-FEV_1_ (*r* = − 0.22, *p* = 0.02), DLCO (*r* = − 0.29, *p* = 0.00) and DLCO% (*r* = − 0.23, *p* = 0.01). Severity scores of cough correlated with pre-FEV_1_ (*r* = − 0.24, *p* = 0.01). Severity scores of sputum correlated with pre-FEV_1_ (*r* = − 0.21, *p* = 0.02). Meanwhile, Severity scores of CAT were correlated with pre-FEV_1_ (*r* = − 0.35, *p* = 0.00) and FEV_1_/FVC (*r* = − 0.27, *p* = 0.00), and CCQ were correlated with pre-FEV_1_ (*r* = − 0.39, *p* = 0.00) and FEV_1_/FVC (*r* = − 0.37, *p* = 0.00). We did not find any associations between chest tightness scores and pulmonary function index (Table 3).

**Table 3:** Correlation between pulmonary functions and scale scores in pneumoconiosis patients with the complication of COPD

	Pre-FEV_1_	FEV_1_/FVC	DLCO	DLCO%
Cough	−0.24(0.01)**	− 0.18(0.06)	− 0.10(0.29)	−0.11(0.22)
Sputum	−0.21(0.02)*	− 0.13(0.16)	− 0.00(0.99)	− 0.04(0.67)
Wheezing	− 0.41(0.00)**	− 0.28(0.00)**	− 0.16(0.08)	− 0.22(0.02)*
Dyspnea	− 0.22(0.02)*	− 0.13(0.16)	− 0.29(0.00)**	− 0.23(0.01)*
Chest tightness	− 0.13(0.17)	− 0.08(0.42)	−0.02(0.83)	− 0.02(0.80)
CAT	−0.35(0.00)**	− 0.27(0.00)**	−0.16(0.08)	− 0.16(0.08)
CCQ	−0.39(0.00)**	− 0.37(0.00)**	−0.14(0.14)	− 0.15(0.10)

Spearman correlation

*p<0.05, **p<0.01

The factors associated with the presence of COPD were analyzed using logistic regression. The factors associated with the presence of COPD were analyzed using logistic regression. In the whole population, in the univariate logistic regression model, pneumoconiosis category (OR, 3.29 [95% CI, 2.49–4.35] per unit higher category; *p* = 0.00), the presence of wheeze symptoms (OR, 2.84 [95% CI, 1.74–4.60]; *p* = 0.00), and age (OR, 2.31 [95% CI, 1.68–3.19] per unit older age; *p* = 0.00) were independent determinants of COPD; In the multivariate logistic regression model, the following factors were independent determinants of COPD: pneumoconiosis category III compared with category II (OR, 10.92 (6.20,19.24) [95% CI, 6.21–19.26]; *p* = 0.00), pneumoconiosis category II compared with category I (OR, 4.39 [95% CI, 2.29–8.41]; *p* = 0.00), the presence of wheeze symptoms (OR, 2.94 [95% CI, 1.79–4.83]; *p* = 0.00), age (OR, 0.90 [95% CI, 0.87–0.93] per 1-year-older age; *p* = 0.00) and reduction in BMI (OR, 1.09 [95% CI, 1.00–1.18] per 1 m^2^/kg reduced BMI; *p* = 0.04) (Table 4).

**Table 4:** OR with 95% CI for the development of COPD in pneumoconiosis patients according to multiple logistic regression analysis

Variable	Univariate analysis		Multivariate analysis	
	OR(95%CI)	*P* value	OR(95%CI)	*P* value
Age	2.31(1.68,3.19)	0.00**	0.90(0.87,0.93)	0.00**
BMI	0.83(0.60,1.17)	0.29	1.09(1.00,1.18)	0.043*
Smoking	1.01(0.91,1.32)	0.32	0.99(0.98,1.01)	0.30
Exposure time	1.18(0.91,1.54)	0.21	0.99(0.96,1.02)	0.44
Air drill type	1.46(0.90,2.37)	0.12	1.38(0.84,2.26)	0.21
Exposure type	1.13(0.61,2.07)	0.70	1.29(0.68,2.45)	0.43
Category	3.29(2.49,4.35)	0.00**	10.92(6.20,19.24)	0.00**
			4.39(2.29,8.41)	0.00**
Cough	1.73(0.42,7.12)	0.45	1.76(0.42,7.38)	0.44
Sputum	0.51(0.14,1.82)	0.30	0.50(0.14,1.79)	0.29
Wheezing	2.84(1.74,4.60)	0.00**	2.94(1.79,4.83)	0.00**
Dyspnea	1.08(0.37,3.13)	0.89	1.12(0.38,3.34)	0.83
Chest tightness	0.98(0.51,1.90)	0.96	0.93(0.48,1.82)	0.83

In univariate analysis model:

Age (1 = 21–30,2 = 31–40,3 = 41–50,4 = 51–60,5 = 61–70,6 = 71–80)

BMI (1 = Below normal, 2 = Normal, 3 = Overweight, 4 = Obesity)

Smoking (1 = Never smokers, 2 = Mild smokers, 3 = Moderate smokers, 4 = Heavy smokers)

Exposure time (1 = 0–10, 2 = 11–20, 3 = 21–30, 4 = 31–40)

Air drill type (1 = No, 2 = Yes)

Exposure type (1 = Silicosis, 2 = Coal)

Category (1 = I, 2 = II, 3 = III)

Cough: 1 = No, 2 = Yes, Sputum: 1 = No, 2 = Yes, Wheezing: 1 = No, 2 = Yes, Dyspnea: 1 = No, 2 = Yes,

Chest tightness:1 = No, 2 = Yes

In multivariate analysis, age, BMI, exposure time and SI were taken as covariate

*OR* odd ratio, *CI* confidence interval

*p<0.05, **p<0.01

However, in pneumoconiosis patients with smoking history, we also found pneumoconiosis category, old age, the presence of wheeze or sputum symptoms were significant risk of COPD in the univariate analysis. Neither type of smoking history or smoking index was a risk of COPD in pneumoconiosis patients with smoking history in the univariate or multivariate analysis (Table 5). In pneumoconiosis patients without smoking history, in the multivariate logistic regression model, pneumoconiosis category III compared with category II (OR, 10.96 [95% CI, 3.50–34.25]; *p* = 0.00), pneumoconiosis category II compared with category I (OR, 10.00 [95% CI, 1.67–60.13]; *p* = 0.01), age (OR, 0.92 [95% CI, 0.86–0.99] per 1-year-older age; *p* = 0.02) were independent determinants of COPD; in the univariate logistic regression model, only pneumoconiosis category (OR, 3.34 [95% CI, 1.88–5.93] per unit higher category; *p* = 0.00) is independent determinants of COPD (Table 6).

**Table 5:** OR with 95% CI for the development of COPD in pneumoconiosis patients with smoking history according to multiple logistic regression analysis

Variable	Univariate analysis		Multivariate analysis	
	OR(95%CI)	*P* value	OR(95%CI)	*P* value
Age	2.55(1.73,3.75)	0.00**	0.89(0.86,0.93)	0.00**
BMI	0.76(0.52,1.12)	0.16	1.09(1.00,1.20)	0.06
Smoking	1.01(0.77,1.56)	0.63	1.00(0.98,1.01)	0.50
Exposure time	1.07(0.78,1.45)	0.68	1.00(0.97,1.03)	0.98
Air drill type	1.51(0.86,2.66)	0.15	1.44(0.81,2.56)	0.22
Exposure type	1.08(0.52,2.25)	0.84	1.22(0.57,2.60)	0.61
Category	3.40(2.43,4.76)	0.00**	11.85(5.97,23.49)	0.00**
			3.92(1.90,8.07)	0.00**
Cough	4.29(0.81,22.65)	0.09	4.16(0.76,22.93)	0.10
Sputum	0.21(0.05,0.95)	0.04*	0.21(0.05,1.00)	0.06
Wheezing	3.00(1.70,5.31)	0.00**	3.10(1.72,5.56)	0.00**
Dyspnea	0.75(0.22,2.53)	0.64	0.75(0.22,2.55)	0.64
Chest tightness	0.94(0.43,2.05)	0.87	0.91(0.41,2.00)	0.81

In univariate analysis model:

Smoking (1 = Mild smokers, 2 = Moderate smokers, 3 = Heavy smokers)

*OR* odd ratio, *CI* confidence interval

*p<0.05, **p<0.01

**Table 6:** OR with 95% CI for the development of COPD in pneumoconiosis patients without smoking history according to multiple logistic regression analysis

Variable	Univariate analysis		Multivariate analysis	
	OR(95%CI)	*P* value	OR(95%CI)	*P* value
Age	1.83(0.99,3.37)	0.05	0.92(0.86,0.99)	0.02*
BMI	1.05(0.52,2.12)	0.90	1.08(0.90,1.29)	0.41
Exposure time	1.64(0.92,2.91)	0.09	0.94(0.88,1.00)	0.05
Air drill type	1.23(0.47,3.23)	0.67	1.02(0.38,2.78)	0.97
Exposure type	1.35(0.40,4.60)	0.63	1.29(0.35,4.77)	0.70
Category	3.34(1.88,5.93)	0.00**	10.96(3.50,34.25)	0.00**
			10.00(1.67,60.13)	0.01**
Cough	0.00(0.00–0.00)	1.00	0.00(0.00–0.00)	1.00
Sputum	0.00(0.00–0.00)	1.00	0.00(0.00–0.00)	1.00
Wheezing	2.40(0.91,6.30)	0.08	2.49(0.90,6.92)	0.08
Dyspnea	5.04(0.38,66.58)	0.22	8.81(0.61,128.08)	0.11
Chest tightness	0.93(0.23,3.76)	0.92	0.79(0.19,3.31)	0.75

*OR* odd ratio, *CI* confidence interval

*p<0.05, **p<0.01

## Discussion

In the current study, we found that the prevalence of COPD was 18.65% in pneumoconiosis. The prevalence of COPD in pneumoconiosis in our study was higher than in the average population [18–21]. A large-population, spirometry-based, cross-sectional survey of COPD prevalence in China in 2007 showed that the overall prevalence of COPD was 12.4% in men and 5.1% in women [18]. Another retrospective study showed that the overall prevalence rate of COPD among Chinese population aged over 40 years in 2013 was 7.3%, and reached as high as 15.5% in the elderly aged over 80 years [19]. In England, population-based study showed the prevalence of spirometrically-defined COPD between 2002 and 2004 was 10% [20]. Whereas it was reported that the prevalence of chronic bronchitis is 18.1% in former coal miners in Ukrainian [20]. And plenty studies demonstrated that the presence of COPD was significantly associated with occupational exposures [9, 20, 22], it was estimated that 10–15% of the total burden of COPD is associated with workplace exposures to dusts, noxious gases/vapors, and fumes [22–25].

We also found that this high prevalence was mainly associated with older age and advanced pneumoconiosis category. Age is often listed as a risk factor for COPD. It is unclear whether healthy aging leads one to be sensitive to COPD or if age reflects the sum of cumulative dust exposures. Nevertheless, the prevalence of COPD increased steeply with age groups and is appreciably high in those over 40 years of age in our study. In addition to age, those with a high pneumoconiosis severity also showed an overwhelming risk of developing COPD, with the highest prevalence among those in pneumoconiosis category III. Pneumoconiosis category is determined by radiographic changes in lung, and the radiographic abnormalities are closely associated with pulmonary function and prognosis in workers exposed to occupational dust [26, 27]. In predominantly active coal miners, decrements in FEV_1_, FVC, and FEV_1_/FVC ratios were greater with the increasing profusion of small opacities [27]. It is worth mentioning that radiographic small opacities may be less prominent as emphysema progresses, and moreover, emphysema due to coal mine dust may occur in the absence of radiographic evidence of CWP [26, 28].

Although we found a significant difference in the distribution of COPD in groups with different exposure times and in the average exposure times between the combined COPD and non-COPD group, the exposure time did not significantly contribute to the risk of COPD in the logistic analysis. These results are similar to those of other studies demonstrating that the duration of silica exposure had no independent effects on lung function [29]. The particularly small population of patients exposed for 31–40 years (51 cases) may also influence the results. Patients less susceptible to developing COPD due to occupational exposure may be those exposed for a rather long period of time (longer than 31 years). In the patients exposed for 31–40 years, we didn’t find any association with the risk of COPD regards to age, pneumoconiosis category, BMI, SI, respiratory symptoms, exposure type or air drill type in the multivariate analysis. In our study, 26.96% of the patients had been suffering from occupational dusts for less than 10 years, and the prevalence of COPD in these patients still reached up to 12.72%. For these “short time” exposed patients, we found only pneumoconiosis category were associated with COPD in the univariate analysis.

In our observations, 73.82% of those with pneumoconiosis were smokers and 26.18% were never-smokers. Pneumoconiosis without smoking history still have a rather high prevalence of COPD and there were no significant difference in COPD prevalence between patients with and without smoking history (91/471, 19.32% vs 28/167, 16.77%, *p* = 0.47). It is well known that dust exposure is associated with both emphysema and airflow obstruction in non-smoking subjects. In Sweden, prevalence of COPD among never-smokers was 3.0–7.7% depending on definition and occupational exposure to gas, dust or fumes was significantly associated with COPD in never-smokers [30]. In one hospital-based cross-sectional study in Turkey, the prevalence of emphysema in non-smoking subjects with CWP was 15% [31]. Another autopsied study on coal miners in the United States showed that the average prevalence of emphysema in non-smoking miners was 30% [32]. In a review of occupational exposure and COPD, the population attributable risk (PAR) for COPD attributable to work was estimated to be 40% in never-smokers and 15% in the overall population [33]. Longitudinal studies have shown that exposure to coal dust has a rapidly decreasing effect on FEV_1_, independent of cigarette smoking [34]. And the progressive massive fibrosis grade and emphysema index at CT were found to be the best independent determinants of FEV_1_,FEV_1_/FVC, and TLC in silicosis. Neither duration of silica exposure nor cigarette consumption had an independent influence on the lung function or clinical parameters, with the exception that cigarette consumption affected DLCO [29]. Because smoking is recognized as an undisputed risk factors risk factor of COPD, we stratified the smoking factor according to smoking history. However, in pneumoconiosis patients with smoking history, neither smoking index nor smoking history type were significant risk of COPD. Advanced category of pneumoconiosis, old age and the presence of wheeze symptoms were the determinant factor of development of COPD in the pneumoconiosis patients. However, the relative small population of patients without smoking history and a healthy smoker effect biasing our results.

Within the COPD group, there were close correlations between lung ventilation function and scores for wheeze, dyspnea, cough, sputum, CAT, CCQ, as well as close correlations between diffusing functions and scores for dyspnea and wheeze. This finding is consistent with the literature report showing that obstructive pulmonary function injury was associated with reported symptoms of dyspnea and wheeze [26]. Pneumoconiosis and COPD may share the same symptoms like cough, sputum, wheezing, dyspnea and chest tightness. However, only the presence of wheeze symptoms and the severity of wheeze and dyspnea were significantly higher in the combined COPD group than in the non-COPD group. This finding reminds us that an inquiry of respiratory symptoms is not enough in the early detection of COPD among patients with pneumoconiosis; instead, pulmonary ventilation function is important in the early screening of COPD [35].

Thus far, there is no curative treatment for pneumoconiosis. It is especially important to delay the onset and slow down the progression. According to current guidelines on the management of COPD, active screening of lung function and smoking cessation [36] is recommended. Prevention measures are critical to decreasing front-line exposures and optimally managing these combined COPD patients among coal or silica-exposed workers. Governments, enterprises, physicians should take effective measures against this situation.

There are some limitations in our study. First, our study is only an institution-based study. In epidemiologic research, population-based studies are necessary for the elimination of the “healthy smokers effect” and the “healthy worker effect”. Second, mineral dust-exposed workers without radiological pneumoconiosis also have exposure-related declines in FEV_1_ and a high prevalence of chronic bronchitis [37, 38]. Therefore, underlying chronic bronchitis cannot be ignored in workers with or without radiological pneumoconiosis, and occupational irritant-induced subclinical lung damage is also worthy of attention. Third, our study did not include female workers because of the extremely low population of female workers in the factories we investigated. Finally, a large population of the patients at the institute are covered by health insurance, which means that there is an unrecognized population of pneumoconiosis patients who are uninsured and probably have a higher prevalence of COPD.

## Conclusions

In summary, we observed that the prevalence COPD is rather high, and advanced pneumoconiosis category, older age were associated with the risk of developing COPD in pneumoconiosis patients. This finding indicates that clinicians must remain vigilant for such COPD-susceptible groups when developing screening protocols for COPD, which will help bring down its prevalence and improve the prognosis. Further prospective cohort studies are needed to confirm these results.
